# FTO, PIK3CB serve as potential markers to complement CEA and CA15-3 for the diagnosis of breast cancer

**DOI:** 10.1097/MD.0000000000035361

**Published:** 2023-10-20

**Authors:** Jintao Mi, Hongsheng Zhang, Weiwei Cao, Chengliang Yuan

**Affiliations:** a College of Medical Technology,Chengdu University of Traditional Chinese Medicine, Chengdu, China; b Department of Clinical Laboratory, People’s Hospital of Deyang City, Deyang, China.

**Keywords:** breast cancer, CA15-3, CEA, diagnostic biomarkers, FTO, PIK3CB

## Abstract

The diagnostic efficacy of carcinoembryonic antigen (CEA) and carbohydrate antigen 15-3 (CA15-3) is limited in breast cancer (BC), highlighting the necessity of exploring novel biomarkers to improve for BC diagnosis. Therefore, we assessed the diagnostic value of fat mass and obesity-associated protein (FTO), phosphatidylinositol-4,5-biphosphate 3-kinase catalytic subunit β (PIK3CB) as a potential complementary biomarker to CEA and CA153 in breast cancer by measuring serum FTO,PIK3CB levels. FTO, PIK3CB, CEA and CA15-3 levels were measured in 112 BC patients and 64 healthy controls using enzyme-linked immunosorbent assay or electrochemiluminescence immunoassay. Spearman’s rank correlation analysis was conducted to assess the correlation between the levels of the 2 markers. The relationships between FTO, PIK3CB, CEA, CA15-3 and clinical characteristics were evaluated. Receiver operating characteristic curve (ROC) analysis was performed to assess the diagnostic value of FTO, PIK3CB, CEA and CA15-3 of BC. Serum FTO, PIK3CB, CEA and CA15-3 levels were significantly increased in BC. There was no correlation between FTO, PIK3CB and CEA, CA15-3. FTO and PIK3CB demonstrated significant diagnostic performance for breast cancer, with FTO achieving a specificity of 90.63%. The diagnostic performance of 2-four biomarker combinations was significantly superior to individual CEA or CA153, with a combined panel of 4 biomarkers yielding an area under the curve (AUC) of 0.918, sensitivity of 81.25% and specificity of 85.94%. In early-stage breast cancer (I + II), the combination of FTO, PIK3CB, CEA and CA153 yielded an AUC of 0.895, sensitivity of 77.22% and specificity of 85.71%. FTO and PIK3CB can be served as potential biomarkers to complement CEA and CA15-3 for BC diagnosis. Combining FTO, PIK3CB, CEA and CA15-3 improves the diagnostic efficiency of breast cancer.

## 1. Introduction

Based on GLOBOCAN 2020 data, breast cancer (BC) has emerged as the most prevalent malignancy among women, accounting for nearly 2.3 million new cases worldwide and resulting in approximately 685,000 deaths.^[1]^ In China, BC significantly affects women health, with around 430,000 new cases and roughly 125,000 deaths estimated in 2022.^[2]^ Despite substantial advancements in breast cancer diagnosis and treatment, there remain patients who face unfavorable prognoses.^[3]^ Research conducted in several European countries has indicated that the reduction in BC mortality can be attributed to the combined impact of early screening and treatment strategies.^[4]^ Consequently, the early detection of BC holds paramount significance in improving patient outcomes and reducing mortality rates.

Presently, noninvasive imaging techniques such as conventional magnetic resonance imaging, conventional ultrasound, full-field digital mammography, and digital breast tomosynthesis have demonstrated sensitivity in preoperative BC diagnosis.^[5,6]^ However, these methods come with high costs, exhibit limitations in sensitivity and positive predictive value, and rely heavily on radiologists’ expertise.^[7]^ Therefore, these early detection approaches for BC might not be optimal or feasible for the general population. In contrast, serum tumor biomarker testing offers a noninvasive diagnostic methods known for its simplicity and convenience, playing a pivotal role in the early detection of various malignancies.^[8]^ Currently, the extensively studied biomarkers for BC are CEA and CA15-3.^[9]^ Nevertheless, the role of CEA and CA15-3 remains contentious due to their limited sensitivity and specificity, especially in early-stage BC.^[9,10]^ Consequently, researchers are actively exploring novel biomarkers to improve the early detection of BC.

In this work, we detected FTO, PIK3CB, CEA, and CA15-3 levels of 112 BC patients and 64 healthy controls. Then, we evaluated the diagnostic value of FTO, PIK3CB, CEA, and CA15-3 alone and in combination for breast cancer.

## 2. Methods

### 2.1. Patients and specimens

This study obtained serum specimens from 112 female breast cancer patients and 64 healthy female individuals who were treated at Deyang People Hospital between September 2021 to May 2023. Serum samples were collected from breast cancer patients either prior to any treatment along with the acquisition of relevant clinical characteristics. The healthy control group was a healthy female physical examination population without any underlying disease. The serum samples were promptly preserved at -80 °C. The American Joint Committee on Cancer Tumor-Node-Metastasis (TNM) classification system was employed for breast cancer staging. The mean age of the breast cancer patients was 51.67 (range 28–84 years). The mean age of the healthy controls was 41.42 (range 23–59 years). The research was authorized by the Ethics Committee of Deyang People Hospital.

### 2.2. Measurement of CEA and CA15-3

We utilized the Siemens ADVIA Centaur XPT electrochemiluminescence immunoassay analyzer (Siemens Healthcare Diagnostics Inc., NY) to measure the serum levels of CEA and CA15-3. The measurements were executed following the manufacturer-recommended protocols. All the measurements were conducted in the laboratory of Deyang People Hospital.

### 2.3. Measurement of FTO and PIK3CB

Serum FTO and PIK3CB were assessed using a commercial enzyme-linked immunosorbent assay kits (Jianglai Biotech, Shanghai, China) according to the manufacturer instructions. The absorbance of each well was measured at 450 nm using a microplate reader (DNM-9606, PERLONG, Beijing, China). A standard curve was generated using the concentration and absorbance values of the standard wells, and the concentrations of FTO and PIK3CB in serum samples were calculated from the absorbance values of the corresponding wells.

### 2.4. Statistical analysis

FTO, PIK3CB, CEA and CA15-3 values were expressed as median (interquartile range). The Shapiro–Wilk test is employed to assess the normality of the data (Supplementary Table 1, http://links.lww.com/MD/K37). Differences between groups were tested using the Mann–Whitney *U* test (difference in 2 groups) or the Kruskal–Wallis test (difference in more than 2 groups). Spearman rank correlation analysis was conducted to assess the correlation between the levels of the 2 markers. Binary logistic regression analysis was used to assess the relationship between marker levels and clinical characteristics. The receiver operating characteristic curves (ROC) evaluated the diagnostic value of each marker alone or in combination. The area under the curve (AUC) was compared using Z tests. Graphs were plotted using SPSS 26.0 (IBM, Armonk, NY) and GraphPad Prism 8 (GraphPad Software, San Diego, CA) analysis. Statistical significance was defined as *P* < .05.

## 3. Results

### 3.1. Serum FTO, PIK3CB, CEA, and CA15-3 levels in BC

**Figure 1:**
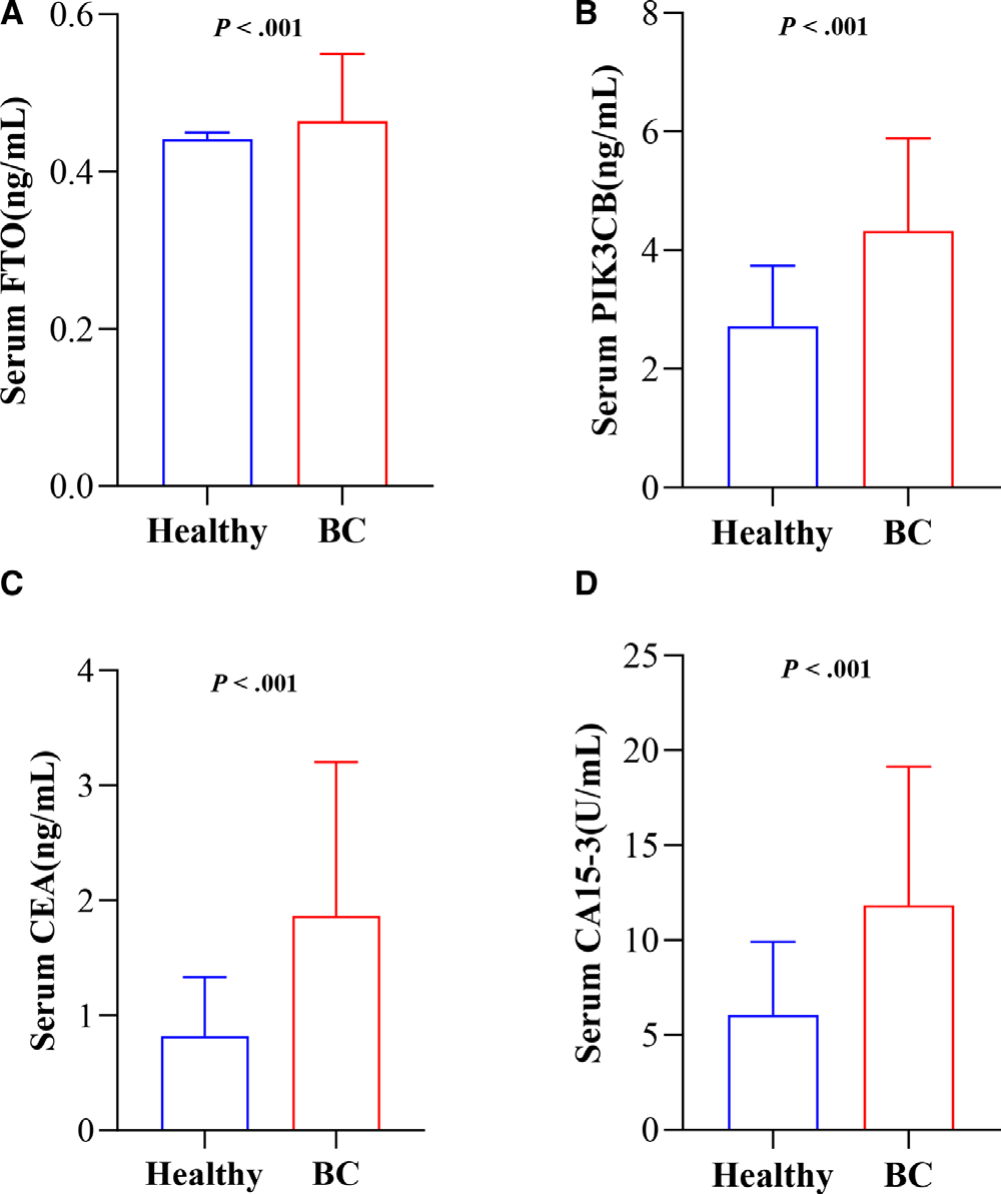
FTO, PIK3CB, CEA, and CA15-3 levels in BC Patients and Healthy controls. FTO (A) and PIK3CB (B) levels were measured using ELISA. CEA (C) and CA15-3 (D) levels were determined by electrochemiluminescence immunoassay. BC = breast cancer, CA15-3 = carbohydrate antigen 15-3, CEA = carcinoembryonic antigen, ELISA = enzyme-linked immunosorbent assay, FTO = fat mass and obesity-associated protein, PIK3CB = phosphatidylinositol-4,5-biphosphate 3-kinase catalytic subunit β.

In BC patients group, the serum levels of FTO (0.464 [0.439, 0.550] ng/mL) and PIK3CB (4.325 [3.310, 5.885] ng/mL) were found to be remarkably increased compared to healthy controls (*P* < .001; Figure 1A and B, Supplementary Table 2, http://links.lww.com/MD/K38). Similarly, the serum levels of CEA (1.865 [1.255, 3.200] ng/mL) and CA15-3 (11.850 [8.225, 19.125] U/mL) were also considerably higher in breast cancer patients group (*P* < .001; Fig. 1C and D).

**Figure 2:**
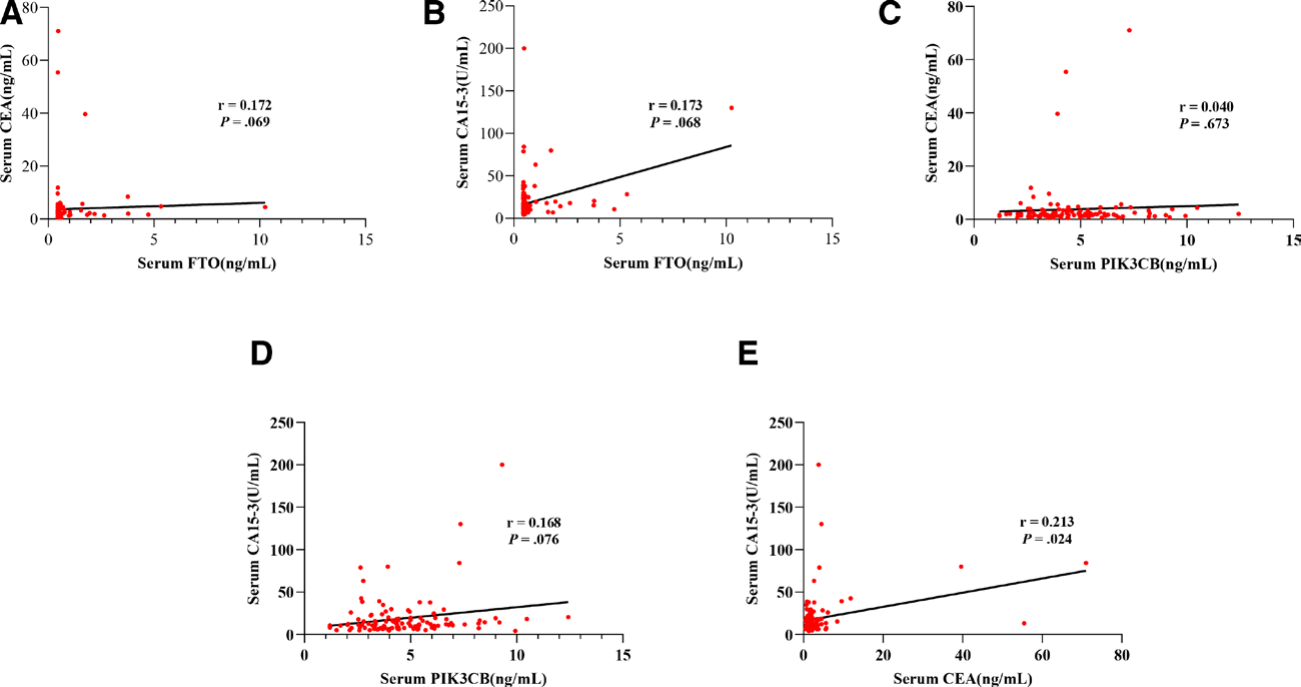
Correlations of serum FTO, PIK3CB, CEA and CA15-3. (A) The correlation among FTO and CEA. (B) The correlation among FTO and CA15-3. (C) The correlation among PIK3CB and CEA. (D) The correlation among PIK3CB and CA15-3. (E) The correlation among CEA and CA15-3. CA15-3 = carbohydrate antigen 15-3, CEA = carcinoembryonic antigen, FTO = fat mass and obesity-associated protein, PIK3CB = phosphatidylinositol-4,5-biphosphate 3-kinase catalytic subunit β.

To assess the relationship between FTO, PIK3CB, CEA, and CA15-3 in BC, we utilized Spearman’s rank correlation analysis. As shown in Figure 2, no correlation was detected between serum FTO, PIK3CB and CEA, CA15-3. These outcomes indicated that FTO, PIK3CB may have a complementary role to CEA and CA153 for the diagnosis of BC.

### 3.2. Association between FTO, PIK3CB, CEA, CA15-3, and clinicopathological features in BC

The association between serum FTO, PIK3CB, CEA, and CA15-3 levels with clinicopathological features is summarized in Tables 1 and 2. The results indicated that FTO levels were significantly associated with tumor TNM stage (*P = *.001), tumor size (*P = *.009) and lymph node metastasis (*P = *.002). Similarly, PIK3CB levels displayed a remarkable association with molecular subtypes (*P* = .036). Furthermore, CEA levels exhibited associations with lymph node metastasis (*P* = .028) and molecular subtypes (*P* = .024). CA15-3 levels were observed to be correlated with tumor TNM stage (*P* = .017) and lymph node metastasis (*P* = .006).

**Table 1:** Association between serum FTO and PIK3CB levels with clinicopathological characteristics of BC patients.

Characteristics	N (%)	FTO (ng/mL)	*P*	PIK3CB (ng/mL)	*P*
Age (yr)					
Average (SD)	51.67 (9.749)				
Median (IQR)	52 (47, 57)				
<50	48 (42.9)	0.451 [0.438, 0.489]		4.123 [2.833, 5.898]	
≥ 50	64 (57.1)	0.477 [0.443, 0.606]	.026*	4.346 [3.494, 5.867]	.333*
TNM stage					
I + II	79 (70.5)	0.453 [0.437, 0.500]		4.138 [3.206, 5.703]	
III + IV	33 (29.5)	0.520 [0.452, 1.000]	.001*	4.334 [3.557, 5.972]	.553*
Tumor size					
<5 cm	92 (82.1)	0.454 [0.438, 0.519]		4.162 [3.310, 5.898]	
>5 cm	20 (17.9)	0.502 [0.458, 1.380]	.009*	4.623 [2.892, 5.808]	.958*
Lymph node metastasis					
Yes	63 (56.2)	0.477 [0.443, 0.697]		4.358 [3.594, 6.098]	
No	49 (43.8)	0.451 [0.437, 0.491]	.002*	3.990 [3.048, 5.409]	.173*
Distant metastasis					
Yes	13 (11.6)	0.509 [0.451, 0.751]		4.888 [3.791, 7.574]	
No	99 (88.4)	0.460 [0.438, 0.553]	.086*	4.138 [3.206, 5.703]	.162*
Molecular subtype					
Luminal A	28 (25)	0.449 [0.439, 0.509]		4.993 [3.539, 6.018]	
Luminal B	38 (33.9)	0.464 [0.438, 0.534]		3.989 [2.756, 5.363]	
Her2^+^	21 (18.8)	0.459 [0.438, 0.581]		3.847 [3.221, 4.358]	
Triple negative	25 (22.3)	0.472 [0.445, 1.011]	.631†	5.468 [3.426, 7.500]	.036†

FTO = fat mass and obesity-associated protein, PIK3CB = phosphatidylinositol-4,5-biphosphate 3-kinase catalytic subunit β, TNM = Tumor-Node-Metastasis.

* Mann–Whitney *U* test.

† Kruskal–Wallis test.

**Table 2:** Association between serum CEA and CA15-3 levels with clinicopathological characteristics BC patients.

Characteristics	N (%)	CEA (ng/mL)	*P*	CA15-3 (U/mL)	*P*
Age (y)					
< 50	48 (42.9)	1.790 [1.235, 2.618]		10.700 [8.300, 13.275]	
≥ 50	64 (57.1)	1.880 [1.255, 3.325]	.823*	15.350 [7.975, 21.775]	.03*
TNM stage					
I + II	79 (70.5)	1.710 [1.210, 2.530]		11.200 [7.600, 17.300]	
III + IV	33 (29.5)	2.220 [1.285, 4.185]	.122*	15.400 [10.700, 27.600]	.017*
Tumor size					
<5 cm	92 (82.1)	1.685 [1.218, 2.618]		11.900 [8.225, 18.475]	
>5 cm	20 (17.9)	2.435 [1.573, 4.268]	.05*	11.050 [7.850, 23.525]	.882*
Lymph node metastasis					
Yes	63 (56.2)	2.060 [1.400, 3.370]		13.200 [9.800, 24.800]	
No	49 (43.8)	1.630 [1.150, 2.235]	.028*	10.800 [6.650, 15.500]	.006*
Distant metastasis					
Yes	13 (11.6)	3.340 [1.270, 5.240]		14.500 [8.350, 37.900]	
No	99 (88.4)	1.790 [1.250, 2.630]	.076*	11.700 [7.900, 18.100]	.192*
Molecular subtype					
Luminal A	28 (25)	2.065 [1.608, 3.758]		11.050 [6.950, 19.100]	
Luminal B	38 (33.9)	2.015 [1.503, 3.758]		11.550 [7.425, 19.750]	
Her2^+^	21 (18.8)	1.400 [0.902, 2.910]		14.200 [11.100, 19.050]	
Triple negative	25 (22.3)	1.410 [1.075, 2.210]	.024†	11.400 [8.600, 19.400]	.578†

CA15-3 = carbohydrate antigen 15-3, CEA = carcinoembryonic antigen, TNM = Tumor-Node-Metastasis.

* Mann–Whitney *U* test.

† Kruskal–Wallis test.

### 3.3. logistic regression analysis

In addition, we conducted logistic regression analysis to assess the relationship between FTO, PIK3CB, CEA, CA15-3 levels and the clinicopathological features of BC. Our results revealed that tumor staging exhibited an independent influence on FTO levels (Table 3). Lymph node metastasis was identified as an independent factor affecting CA15-3 levels. However, our results showed that neither CEA nor PIK3CB levels were affected by tumor stage, tumor size, lymph node metastasis or distant metastasis.

**Table 3:** Logistic regression analysis of markers and clinicopathological features in BC.

Characteristics	FTO			PIK3CB			CEA			CA15-3		
	β	Exp (B)	*P*	β	Exp (B)	*P*	β	Exp (B)	*P*	β	Exp (B)	*P*
Age	0.406	1.502	.166	0.084	1.087	.379	−0.019	0.981	.399	0.003	1.003	.709
TNM stage	−0.599	0.549	.028	−0.057	0.945	.565	−0.011	0.989	.604	−0.02	0.98	.056
Tumor size	0.386	1.471	.054	0.023	1.023	.845	0.019	1.019	.393	0.015	1.015	.087
Lymph node metastasis	1.022	2.778	.068	0.132	1.141	.176	0.135	1.144	.226	0.049	1.051	.024
Distant metastasis	0.100	1.105	.611	0.197	1.218	.124	0.03	1.031	.171	0.016	1.016	.071

CA15-3 = carbohydrate antigen 15-3, CEA = carcinoembryonic antigen, FTO = fat mass and obesity-associated protein, PIK3CB = phosphatidylinositol-4,5-biphosphate 3-kinase catalytic subunit β, TNM = Tumor-Node-Metastasis.

### 3.4. Diagnostic value of FTO, PIK3CB, CEA, and CA15-3 in BC

**Figure 3:**
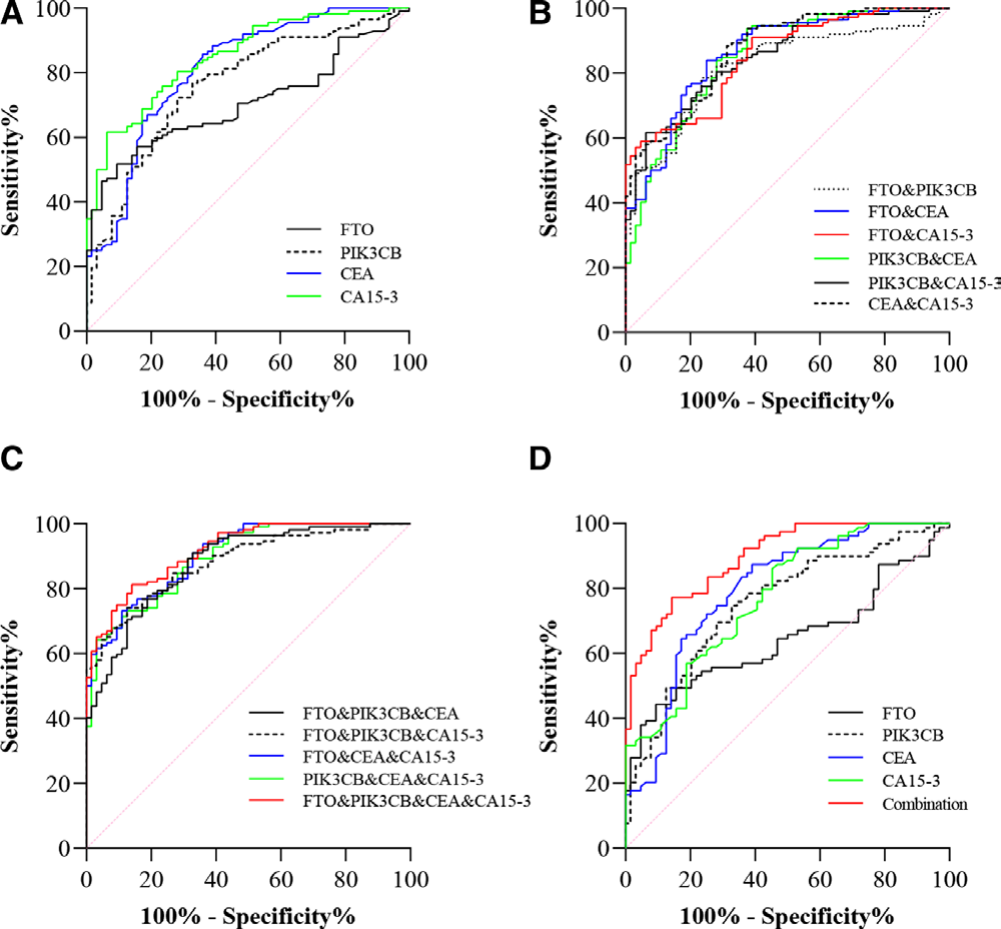
Diagnostic value of markers in BC. (A) Diagnostic value of a single marker in BC. (B) Diagnostic value of the combination of 2 markers in BC. (C) Diagnostic value of the combination of 3 or 4 markers in BC. (D) Diagnostic value of FTO, PIK3CB, CEA, CA15-3 Alone and in Combination for Early-Stage BC. BC = breast cancer, CA15-3 = carbohydrate antigen 15-3, CEA = carcinoembryonic antigen, FTO = fat mass and obesity-associated protein, PIK3CB = phosphatidylinositol-4,5-biphosphate 3-kinase catalytic subunit β.

The diagnostic value of FTO, PIK3CB, CEA and CA15-3 in breast cancer was evaluated by constructing ROC curves. These 4 biomarkers demonstrated significant diagnostic value in distinguishing BC from healthy controls (AUC_FTO_ = 0.705 [0.630–0.780], AUC_PIK3CB_ = 0.762 [0.689–0.835], AUC_CEA_ = 0.810 [0.743–0.878], AUC_CA15-3_ = 0.801 [0.736–0.867], Table 4 and Fig. 3A).

**Table 4:** Performance of markers in the diagnosis of BC.

Tumor markers	AUC (95%CI)	Cutoff value	Sensitivity (%)	Specificity (%)	*P*
FTO	0.705 (0.630–0.780)	0.462	51.79%	90.63%	-
PIK3CB	0.762 (0.689–0.835)	3.314	75.00%	67.19%	-
CEA	0.810 (0.743–0.878)	1.105	85.71%	63.49%	-
CA15-3	0.801 (0.736–0.867)	10.250	64.29%	80.95%	-
FTO + PIK3CB	0.821 (0.759–0.883)	-	78.57%	76.56%	-
FTO + CEA	0.862 (0.806–0.917)	-	83.93%	75.00%	.006*
FTO + CA15-3	0.849 (0.794–0.904)	-	58.04%	95.31%	.016†
PIK3CB + CEA	0.849 (0.790–0.908)	-	93.75%	62.50%	-
PIK3CB + CA15-3	0.849 (0.793–0.905)	-	61.61%	93.75%	.033†
CEA + CA15-3	0.869 (0.818–0.921)	-	88.39%	68.75%	.018*, .006†
FTO + PIK3CB + CEA	0.879 (0.829–0.929)	-	91.07%	67.19%	.004*
FTO + PIK3CB + CA15-3	0.883 (0.835–0.930)	-	74.11%	87.50%	.002†
FTO + CEA + CA15-3	0.904 (0.861–0.946)	-	73.21%	89.06%	.001*, <.001†
PIK3CB + CEA + CA15-3	0.895 (0.850–0.940)	-	64.29%	96.88%	.002*, .001†
FTO + PIK3CB + CEA + CA15-3	0.918 (0.879–0.956)	-	81.25%	85.94%	<.001*, <.001†

AUC = area under the curve, CA15-3 = carbohydrate antigen 15-3, CEA = carcinoembryonic antigen, FTO = fat mass and obesity-associated protein, PIK3CB = phosphatidylinositol-4,5-biphosphate 3-kinase catalytic subunit β.

* In comparison with CEA.

† In comparison with CA15-3.

As displayed in Table 4, it was determined that the optimal cutoff values for FTO, PIK3CB, CEA and CA15-3 were based on the Youden’s index. In differentiating between BC and healthy controls, the optimal cutoff value for FTO was 0.462 ng/mL, with a sensitivity of 51.79% and specificity of 90.63%. Notably, FTO exhibited the highest specificity among the individual biomarkers. For PIK3CB, the optimal cutoff value was 3.314 ng/mL, with a sensitivity of 75.00% and specificity of 67.19%. Regarding CEA, the optimal cutoff value was 1.105 ng/mL, with a sensitivity of 85.71% and specificity of 63.49%. Lastly CA15-3, the optimal cutoff value was 10.250 U/mL, with a sensitivity of 64.29% and specificity of 80.95%.

To evaluate whether FTO and PIK3CB enhance the discriminative capability of CEA and CA15-3, we compared the diagnostic performance of various marker combinations with that of using CEA or CA15-3 individually. As illustrated in Table 4 and Figure 3B, the combined AUC of FTO or PIK3CB with CEA or CA15-3 exceeded that of CEA or CA15-3 alone. The utilization of 2 combined markers led to an enhanced sensitivity (93.75%) or specificity (95.31%). As illustrated in Table 4 and Figure 3C, the AUC of the 3 markers combined was apparently higher than that of CEA or CA15-3 alone. Moreover, the combined AUC of the 4 markers was remarkable greater than that of CEA or CA15-3 alone, with sensitivities and specificities reaching 81.25% and 85.94%, respectively.

### 3.5. FTO and PIK3CB in the diagnosis of early-stage breast cancer

By analyzing the clinicopathological features, we found that FTO and CA15-3 are associated with TNM stage. For early-stage BC patients (Stage I + II, n = 79), we assessed the diagnostic value of FTO, PIK3CB, CEA and CA15-3 (AUC_FTO_ = 0.646 [0.556–0.737], AUC_PIK3CB_ = 0.751 [0.670–0.831], AUC_CEA_ = 0.792 [0.716–0.868], AUC_CA15-3_ = 0.772 [0.696, 0.848], Table 5 and Fig. 3D). Compared with using CEA or CA15-3 alone, the combination of 4 markers showed significantly higher AUC values in the diagnosis of early-stage BC.

**Table 5:** Performance of markers in the diagnosis of early-stage BC.

Tumor markers	AUC(95%CI)	Cutoff value	Sensitivity (%)	Specificity (%)	*P*
FTO	0.646 (0.556–0.737)	0.463	44.30%	90.63%	-
PIK3CB	0.751 (0.670–0.831)	3.247	74.68%	66.67%	-
CEA	0.792 (0.716–0.868)	1.025	87.34%	60.32%	-
CA153	0.772 (0.696–0.848)	6.350	86.08%	55.56%	-
Combination	0.895 (0.846–0.944)	-	77.22%	85.71%	.001*, <.001†

AUC = area under the curve, CEA = carcinoembryonic antigen, FTO = fat mass and obesity-associated protein, PIK3CB = phosphatidylinositol-4,5-biphosphate 3-kinase catalytic subunit β.

* In comparison with CEA.

† In comparison with CA15-3.

## 4. Discussion

Breast cancer is characterized by highly heterogeneous.^[11]^ Currently, there is a persistent rise in reported cases of BC among young women, with low 5-year survival rates, which have been associated with delayed diagnosis and higher local recurrence rates.^[12]^ In the early-stages of BC, commonly utilized markers like CEA and CA15-3 demonstrate relatively limited sensitivity and specificity.^[13]^ Therefore, in order to facilitate early diagnosis of breast cancer, it is crucial to investigate novel serum markers. Our work identified elevated serum levels of FTO and PIK3CB in BC patients. Moreover, the combination of FTO, PIK3CB, CEA, and CA15-3 exhibited an improved diagnostic value for breast cancer compared to the use of CEA or CA15-3 alone. Thus, the inclusion of FTO and PIK3CB in the diagnostic panel can enhance the diagnostic effectiveness of CEA and CA15-3 in breast cancer.

FTO has been identified firstly as the RNA demethylase, with N6-methyladenosine (m^6^A) as its main substrate.^[14,15]^ m6A is a prevalent internal modification found in messenger RNA (mRNA), and its dysregulation can lead to RNA disruption, thereby influencing tumor development at the transcriptional level.^[16,17]^ Increasing evidence suggests that FTO is highly expressed in leukemia, melanoma, gastric cancer, and cervical cancer.^[17–20]^ Several studies have demonstrated the potential of FTO as a diagnostic and prognostic biomarker in lung cancer and gastric cancer.^[19,21]^ Moreover, previous investigations by Xu et al^[22]^ and Niu et al^[23]^ have suggested that FTO plays a role in BC progression and may hold promise as a diagnostic marker for BC. In this study, we observed an increased expression of serum FTO levels was observed in BC patients. Clinical data analysis exhibited a relationship between FTO and tumor stage in BC patients. Specifically, higher FTO levels were detected in stage III + IV BC compared to stage I + II BC. Furthermore, FTO were also correlated with tumor size, lymph node metastasis. Binary logistic regression analysis identified tumor stage as an independent factor influencing FTO levels in BC. Moreover, we demonstrated the diagnostic value of FTO in BC through ROC curve analysis, revealing an AUC of 0.705 [0.630–0.780], sensitivity of 51.79%, and specificity of 90.63%. FTO exhibits significant diagnostic performance in distinguishing BC from healthy controls.

PIK3CB belongs to the phosphatidylinositol 3-kinase (PI3K) family and is a key molecule in the PI3K signaling pathway.^[24,25]^ Recent studies have shown that elevated PI3K signaling has become a hallmark of human cancers and can promote tumor development and progression.^[26]^ Dysregulation of kinases such as PIK3CB is commonly observed in cancer and is related to tumorigenesis.^[27]^ Research has identified PIK3CB as a biomarker for cancer recurrence and prognosis, including renal clear cell carcinoma, glioblastoma, and oral squamous cell carcinoma.^[28–30]^ In this research, we noticed an increased serum PIK3CB levels in BC patients. Serum PIK3CB levels were associated with molecular subtypes. We evaluated the diagnostic value of PIK3CB in BC, revealing an AUC of 0.762 [0.689–0.835], sensitivity of 75.00% and specificity of 67.19%. PIK3CB also exhibited significant diagnostic performance in differentiating breast cancer from healthy controls.

CEA and CA15-3 are widely utilized in the diagnosis and prognosis of BC.^[31,32]^ This study identified increased CEA and CA15-3 levels in BC patients. Through clinical feature analysis, serum CEA level was linked to lymph node metastasis and molecular subtypes, while serum CA15-3 level was linked to TNM stage and lymph node metastasis. Furthermore, logistic regression analysis identified lymph node metastasis as an independent factor influencing serum CA15-3 levels. To evaluate their diagnostic ability, ROC curve analysis was performed to differentiate breast cancer from healthy controls using serum CEA and CA15-3 levels. CEA exhibited an AUC of 0.810 [0.743–0.878], sensitivity of 85.71% and specificity of 63.49%. While CA15-3 exhibited an AUC of 0.801 [0.736–0.867], sensitivity of 64.29%, and specificity of 80.95%. The results of this study align with previous findings, indicating that the sensitivity and specificity of CEA and CA15-3 in BC diagnosis were suboptimal.^[13]^ Therefore, monitoring CEA and CA15-3 alone may not meet clinical needs. We evaluated the relationship between serum levels of FTO, PIK3CB and CEA or CA15-3, the outcomes demonstrated that FTO and PIK3CB were not correlated with CEA and CA15-3, suggesting their potential as supplementary biomarkers to CEA and CA15-3. Next, we plotted ROC curves to assess the diagnostic value of the 2 biomarkers in combination (Table 4). The diagnostic performance of FTO combined with CEA or CA15-3 was significantly higher than that of CEA or CA15-3 alone. Similarly, the diagnostic performance of PIK3CB combined with CA15-3 was considerably higher than that of CA15-3 alone, with a specificity increase to 93.75%. We also plotted ROC curves for the combination of all 3 biomarkers (Table 4), and the diagnostic performance of the 3 biomarkers combined was greatly higher than that of CEA or CA15-3 alone, with a sensitivity ranging from 64.29% to 91.07% and a specificity ranging from 67.19% to 96.88%. Finally, we plotted an ROC curve for the combination of all 4 biomarkers, and the results showed that the diagnostic performance of the 4 biomarkers combined was the best, with improvements in both sensitivity and specificity. Since FTO and CA15-3 have demonstrated value in early-stage BC, we plotted ROC curves to explore the diagnostic performance of the biomarkers in distinguishing early-stage BC from healthy controls. The results showed that the diagnostic performance of the 4 biomarkers combined was significantly higher than that of monitoring early breast cancer with CEA or CA15-3 alone, with an increased specificity of 85.71%. These findings indicate the importance of combining FTO, PIK3CB, CEA, and CA15-3 in the monitoring of early-stage BC.

There are still some limitations in this paper, such as small sample size and no multi-center validation, which should be focused on in the next step. In addition, we did not investigate the prognostic value of FTO,PIK3CB on breast cancer, so we hope to explore the prognostic significance of FTO,PIK3CB on breast cancer in subsequent experiments.

## 5. Conclusions

This study demonstrated that the combination of FTO, PIK3CB, CEA and CA15-3 improves the diagnostic efficiency of BC. These findings indicate that FTO and PIK3CB have the potential to serve as supplementary markers to complement CEA and CA15-3 for the diagnosis of BC. Moreover, the combination of FTO, PIK3CB, CEA and CA15-3 holds significant value for monitoring early-stage BC.

## Author contributions

**Conceptualization:** Weiwei Cao, Chengliang Yuan.

**Data curation:** Jintao Mi, Hongsheng Zhang.

**Formal analysis:** Hongsheng Zhang, Weiwei Cao.

**Funding acquisition:** Chengliang Yuan.

**Investigation:** Jintao Mi, Hongsheng Zhang.

**Methodology:** Jintao Mi, Hongsheng Zhang.

**Project administration:** Jintao Mi, Hongsheng Zhang.

**Resources:** Jintao Mi, Hongsheng Zhang.

**Software:** Jintao Mi, Hongsheng Zhang.

**Supervision:** Weiwei Cao, Chengliang Yuan.

**Validation:** Jintao Mi, Hongsheng Zhang.

**Visualization:** Jintao Mi, Hongsheng Zhang.

**Writing – original draft:** Jintao Mi, Hongsheng Zhang, Weiwei Cao, Chengliang Yuan.

**Writing – review & editing:** Jintao Mi, Hongsheng Zhang, Weiwei Cao, Chengliang Yuan.

## Supplementary Material




