# Supplementary material for: FTO, PIK3CB serve as potential markers to complement CEA and CA15-3 for the diagnosis of breast cancer
Source: Medicine (Baltimore). 2023 Oct 20;102(42):e35361. doi: 10.1097/MD.0000000000035361 (PMC10589555; doi:10.1097/MD.0000000000035361)
Supplement: Supplementary file 1 [file medi-102-e35361-s001.docx]

**Supplementary table 1**

**Shapiro-Wilk test for marker parameter normality in Healthy controls and BC.**

|  | Healthy | *P* | BC | *P* |
| --- | --- | --- | --- | --- |
| FTO | 0.441[0.438, 0.450] | <0.001 | 0.464[0.439, 0.550] | <0.001 |
| PIK3CB | 2.726[2.215, 3.750] | 0.001 | 4.325[3.310, 5.885] | <0.001 |
| CEA | 0.820[0.320, 1.330] | <0.001 | 1.865[1.255, 3.200] | <0.001 |
| CA-153 | 6.000[4.200, 10.00] | 0.001 | 11.850[8.225, 19.125] | <0.001 |
