# Supplementary material for: FTO, PIK3CB serve as potential markers to complement CEA and CA15-3 for the diagnosis of breast cancer
Source: Medicine (Baltimore). 2023 Oct 20;102(42):e35361. doi: 10.1097/MD.0000000000035361 (PMC10589555; doi:10.1097/MD.0000000000035361)
Supplement: Supplementary file 2 [file medi-102-e35361-s002.docx]

**Supplementary Table 2**

**Serum FTO, PIK3CB, CEA and CA15-3 levels in Healthy controls and BC.**

|  | **Healthy** | **Breast cancer** | ***P*** |
| --- | --- | --- | --- |
| N | 64 | 112 | - |
| FTO (ng/ml) | 0.441[0.438, 0.450] | 0.464[0.439, 0.550] | < .001 |
| PIK3CB (ng/ml) | 2.726[2.215, 3.750] | 4.325[3.310, 5.885] | < .001 |
| CEA (ng/ml) | 0.820[0.320, 1.330] | 1.865[1.255, 3.200] | < .001 |
| CA15-3 (U/ml) | 6.000[4.200, 10.00] | 11.850[8.225, 19.125] | < .001 |
